# Cervicothoracic Manipulation Techniques Reviewed Utilizing Three-Dimensional Spine Model

**DOI:** 10.7759/cureus.5836

**Published:** 2019-10-04

**Authors:** Ryan C McCoy, Edsel Bittencourt, William Clifton

**Affiliations:** 1 Physical Therapy, Mayo Clinic, Jacksonville, USA; 2 Neurosurgery, Mayo Clinic, Jacksonville, USA

**Keywords:** manual therapy, spinal manipulation, learning, 3d-printed spine model

## Abstract

Currently, there is a paucity of studies that describe prone cervicothoracic joint high-velocity low-amplitude (HVLA) thrust techniques using an anatomically accurate, biomimetic three-dimensional (3D) spine model for educational demonstration. The purpose of this technical report was to present a learning model for two prone cervicothoracic HVLA thrust techniques using a 3D model, review intersegmental mobility observed on a 3D spine model with application of the techniques, and lastly discuss potential applications of this learning model.

## Introduction

There has been an increasing application of three-dimensional (3D) spine models to facilitate learning for students and healthcare providers [1]. 3D spine models are being utilized in healthcare to enhance student and practitioner learning for applied pathophysiology and intervention simulation [1]. There is a paucity of studies supporting the use of 3D models to facilitate student and practitioner learning of specific manual therapy intervention techniques. More specifically, there are no studies to date assessing the use of a 3D printed spine as a learning model describing osteokinematics for prone cervicothoracic high-velocity low-amplitude (HVLA) thrust techniques.

Manual therapy techniques have been well described in the literature over the years and date back well beyond the scope of this study [2-12]. The benefits of HVLA thrust techniques for spinal and extremity pain or dysfunction are widely recognized in the literature. For example, reduction in pain and improved function following HVLA thrust manipulation to the cervicothoracic region has been widely reported in patients with neck pain and shoulder pain [2, 5-7, 10]. Achieving isolated specific facet cavitation is not consistent with the literature when applying segment-specific HVLA techniques throughout the upper and lower cervical, cervicothoracic, thoracic, and lumbar spine regions [2-3, 9]. Though joint cavitation at an intended facet may not be as specific as our intention to treat, the application of HVLA techniques at the cervicothoracic spine should be. The lateral break cervicothoracic joint HVLA maneuver has been well described [3]. Utilization of the lateral break maneuver at the cervicothoracic junction produced cavitation that was significantly more likely to occur unilaterally and on the side contralateral to the short lever applicator contact [3]. Variations of this manual technique and the respective intersegmental mobility have not been well described utilizing a 3D spine model.

Manual therapy skills are highly dependent upon the practitioner and their respective years of experience which makes consistent application of the intervention harder to replicate. In the author’s experience, it is also common for healthcare providers and students utilizing manual therapy techniques to report a hand dominance preference when applying a lever arm versus a mobilizing arm for HVLA techniques. These factors could bias HVLA thrust techniques being utilized in clinical practice regardless of patient presentation. This further demonstrates the necessity of reviewing and investigating varying manual therapy techniques to help clinicians optimize applied interventions. Clinical application of HVLA thrust techniques requires strong clinical reasoning based upon the patients' presentation and comfort level, a thorough evaluation with evidence supporting the intended intervention, clinician experience, and patient comfort with the applied techniques. Evidence suggests that adverse effects from thoracic manipulation are likely decreased by performing a thorough exam and using sound clinical judgement [4]. The author would also suggest that in addition to the previously mentioned statement that a consistent and repeatable manipulation intervention approach with high clinician confidence for intention to treat may also reduce adverse effects of joint manipulation. With a high prevalence of neck, thoracic, and shoulder pain this highlights the necessity for improving the efficacy of cervicothoracic HVLA thrust manipulation techniques. Therefore, a technical report and review of two cervicothoracic HVLA thrust techniques utilizing a 3D printed model may be beneficial to educate providers and students for clinical application. The description of ipsilateral and contralateral facet intersegmental mobility will be reviewed utilizing a 3D model along with patient and provider set up. The purpose of this technical report is to (1) present a learning model for two varying prone cervicothoracic HVLA techniques on a 3D model, (2) review intersegmental mobility observed on a 3D spine model with application of two cervicothoracic HVLA thrust techniques, (3) and lastly discuss implication and potential application of this learning model.

## Technical report

**Figure 1:**
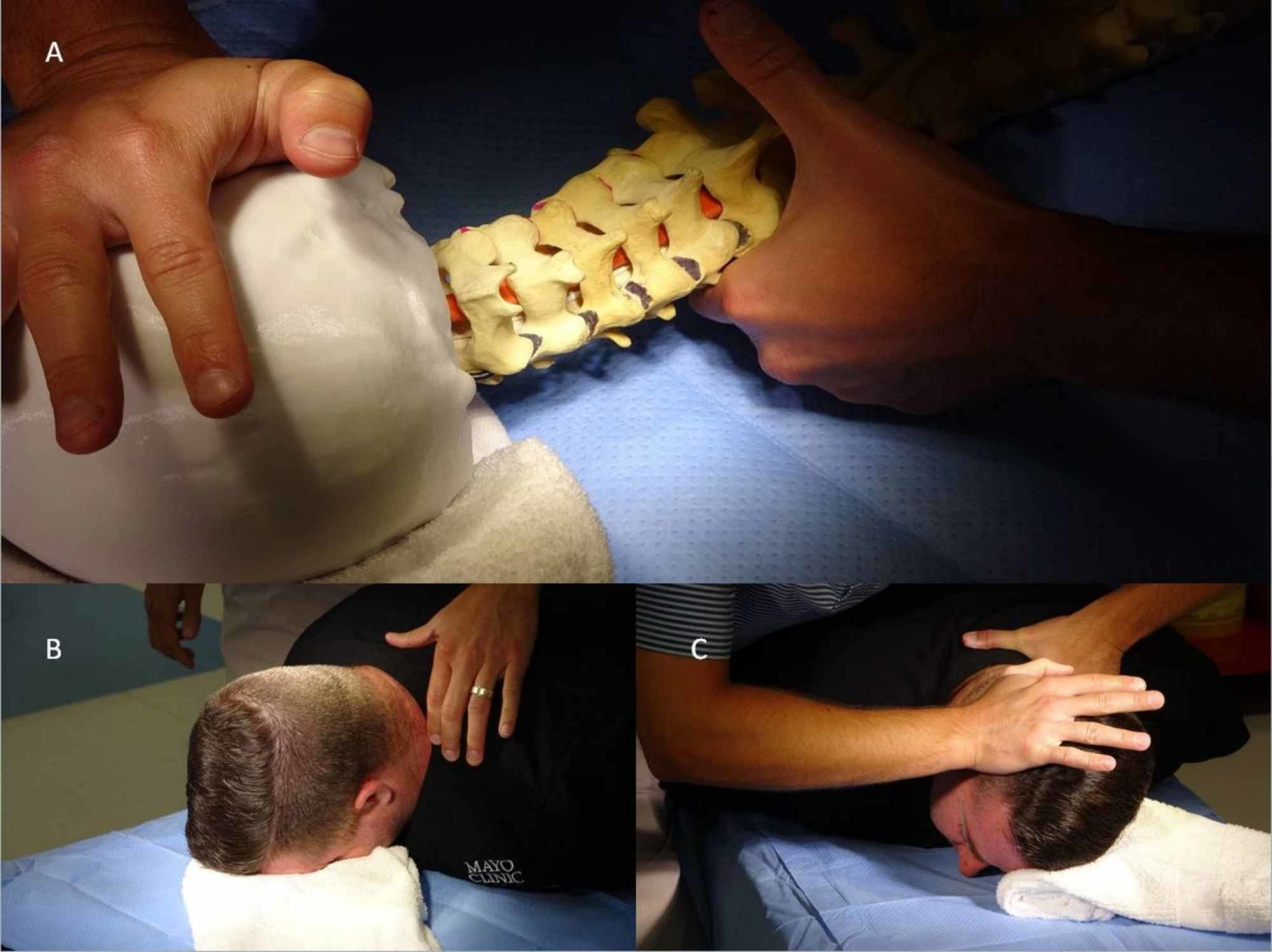
Prone right cervicothoracic manipulation technique (A) Visualization of left facet upglide during prone right cervicothoracic mobilization technique. (B) Palpation of C7 Spinous process with the patient prone in a comfortable position prior to mobilization technique. (C) Set up depicted for prone right cervicothoracic manipulation at C7-T1 with therapist standing to right of patient on a high low table. Left hand point of contact is through second metacarpophalangeal joint at the articular pillar of intended spinal segment and right mobilizing hand at lateral and posterior aspect of head which is supported by a towel roll at the anterior aspect of the subjects right temporal area promoting right rotation up to the cervicothoracic joint junction.

The intention of this learning model is to practice first with the 3D model. Upon student or provider comfort they can then work on a subject if deemed appropriate. The left zygapophyseal joint, or facet joints, were colored in black and the right in pink in order to better visualize mobility at the respective joints (See Figure 1A). Two prone cervicothoracic manual therapy techniques will be reviewed on the 3D spine model. Technique selection between the two maneuvers will vary based upon patient needs, provider preference, and intention of treatment. Rationale for clinical application of each technique will be described. The first technique that will be reviewed is the rotational cervicothoracic HVLA thrust. Utilize a high low table for appropriate body mechanics. Have a towel roll on hand to assist with patient positioning and comfort. Following assessment that warrants cervicothoracic mobilizations begin with the model prone. For clarity, this technique describes the model prone with right cervical rotation. The therapist will be standing on the right side of the table. Stand in a tandem stance with right leg forward angled towards the head of the 3D spine model. Palpate the interspinous process for the intended segment of mobilization, which in this description will be C7-T1 (See Figure 1B). For the rotational cervicothoracic manipulation, the towel roll will support the anterior and lateral aspect of the left temporal and parietal region of the model with the intention to ensure patient comfort (See Figure 1C). Maintaining point of contact at C7-T1 interspinous segment, gently rotate the models head to the right until intersegmental motion is first felt at C7-T1. When working with a patient, they will actively rotate their head. At the palpated level place the medial aspect of the second metacarpophalangeal of the left hand at the left articular pillar targeting C7-T1. The articular pillar is the space occupied by paraspinal musculature which lies between the spinous process and transverse process of the respective spinal segment. Place right hand at the lateral and posterior aspect of the right temporal area just superior and posterior to the subject’s right ear. Utilizing proper body mechanics apply stabilizing leverage through left arm which has a direct medial and slight inferior line of force through the point of contact of metacarpophalangeal joint. Next, apply graded mobilizations through right arm at the point of contact in a lateral and slightly superior direction facilitating right cervicothoracic rotation while maintaining leverage through left hand at C7-T1 articular pillar with intention to assess symptoms, reactivity, and joint end feel. If the HVLA thrust technique is deemed appropriate, apply HVLA thrust technique. This technique promotes intersegmental upglide of left facets and relative downglides of the contralateral right facets at intended area of mobilization (See Figure 1A).

**Figure 2:**
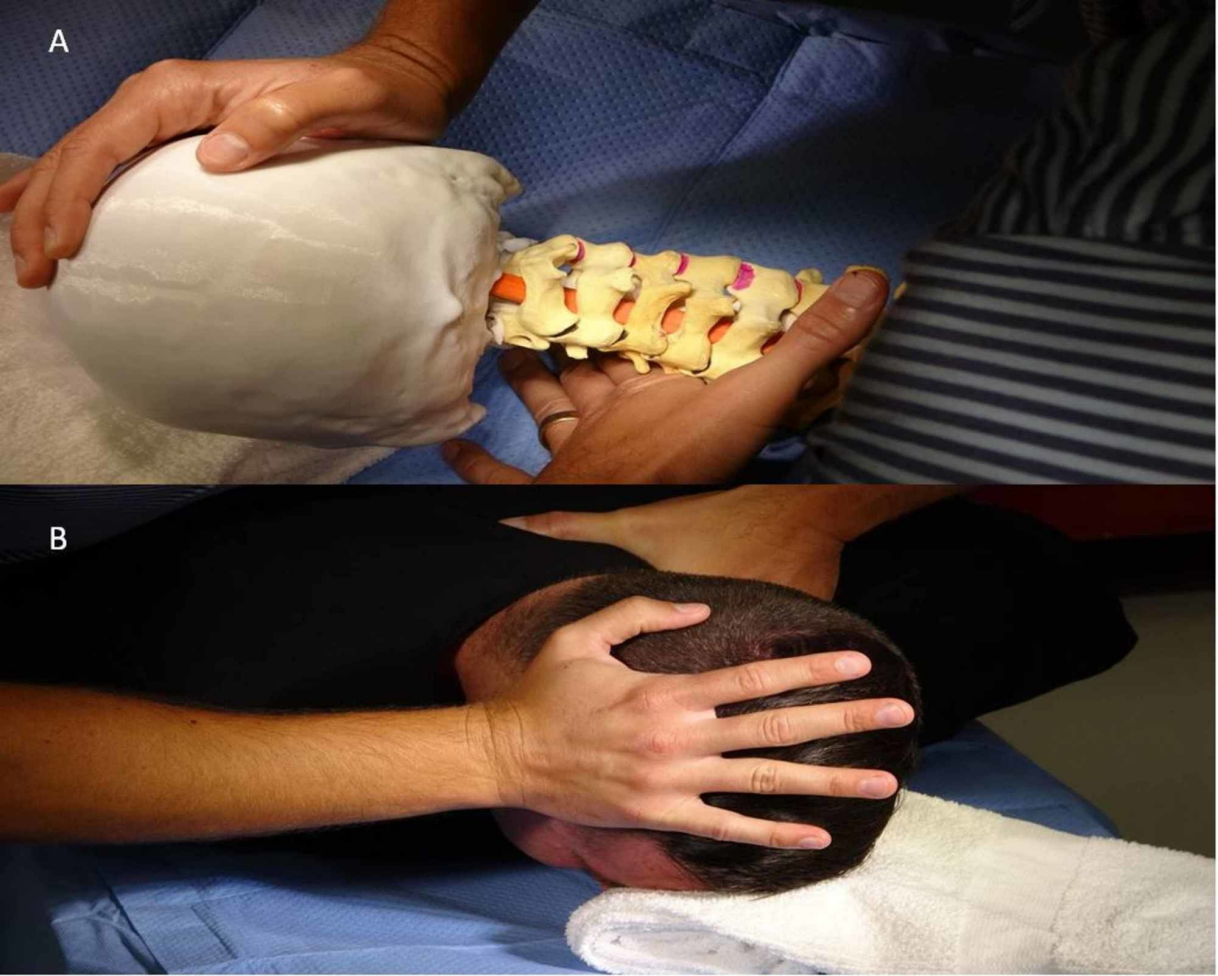
Prone lateral break cervicothoracic maneuver (A) Set up for lateral break cervicothoracic joint HVLA thrust on 3D spine model. Right facet upglide depicted at intended segments during application of lateral break cervicothoracic maneuver promoting left side bending. (B) Set up for lateral break cervicothoracic manipulation technique on subject with therapist standing on right side of the high low table with towel roll support to forehead. Left hand point of contact through second metacarpophalangeal joint at intended segment with line of force in the medial direction and right mobilizing hand to lateral aspect of right temporal region.

The other prone technique is the lateral break method (See Figure 2). The cervicothoracic lateral break method similarly begins with the model prone and palpation at C7-T1 interspinous process as described in the previous technique. Provide a towel roll to support the left side of the patient's head with minimal right cervical rotation or place the towel roll directly supporting the forehead with the cervical spine in a neutral position. Stand in a tandem stance with right leg forward at a slight angle towards the head of the model. Place the medial aspect of the second metacarpophalangeal of the left hand just lateral to the left articular pillar of C7-T1 with a direct medial line of force through the left arm. Place right hand at the superior aspect of the right temporal area just superior to visualized right ear. Apply an opposing medial and slightly superior direction of force through point of contact of right arm inducing left cervicothoracic side bending. Apply a graded force assessing theoretical symptoms, tissue reactivity, patient comfort, and joint end feel. If deemed appropriate apply a HVLA thrust. This technique will promote right cervicothoracic facet upglide and relative downglide at left cervicothoracic facets (see Figure 2A). Contrary to the rotational cervicothoracic HVLA technique, intersegmental mobility varies with the lateral break method. Technique selection can be applied clinically based on the rationale of the provider. 

## Discussion

Practice of manual therapy skills on a 3D spine model may facilitate learning and safety for students and healthcare providers. Visualization of the intersegmental mobility can be observed with application of the described cervicothoracic handling techniques. Patient and provider set up can be reviewed and optimized which may enhance safety prior to the practitioner’s first trial of applying the mobilization technique. Patient position can be alternated to bias cervicothoracic intersegmental mobility based upon the technique being utilized. Further, provider positioning can be tailored if they have a preference for mobilization versus stabilizing lever arm application. Commonly, students and clinicians who are practicing a new HVLA thrust technique will tend to utilize a preferred mobilizing left or right upper extremity. Reviewing these two prone cervicothoracic techniques can help improve intention to treat based on intersegmental mobility desired at the cervicothoracic junction and based on manual preference from the provider if one exists. For example, the primary author has a right-handed mobilization preference. Utilization of these two prone cervicothoracic HVLA techniques facilitates a comfortable provider set up with increased confidence for a relative upglide cervicothoracic thrust maneuver at either the left or right cervicothoracic facet joints utilizing the right upper extremity as the mobilizing arm for either technique. It is in the author’s experience that patients consistently report cavitation felt on the left cervicothoracic facets with the right rotational cervicothoracic HVLA technique and on the right lower facets with the lateral break method promoting left cervicothoracic side bending. Although joint cavitation may not be consistent for all patients using a specific intervention, it is important to still understand facet mobility with applied interventions in order to reduce the risk of generalizing interventions and improving manual rationale.

At a low cost these reviewed manual techniques were able to be performed on a 3D spine model which demonstrated relative intersegmental zygapophyseal joint mobility at the cervicothoracic joint. In a clinical or learner setting this may enhance instruction and efficacy of these specific techniques utilized in practice prior to subject trials. Instructional methods incorporating the use of 3D spine models have been utilized in osteopathic, chiropractic and physical therapy education settings for years. Interestingly, learner performance for application of manipulation on a 3D spine prior to subjects trials has not been well studied. It has not been well described in the literature if manipulation on a 3D model improves the efficacy of learner performance in comparison to immediate practice on live subjects after traditional method instruction. Traditional methods for teaching manual therapy skills can include lecture format, video instruction, and classroom instruction. Classroom instruction can include instructor demonstration on a subject, instructor demonstration on a model, and instructor demonstration on the learner. There is no evidence of the author's knowledge addressing learner performance when applying the reviewed manipulation techniques on a 3D model prior to subject trials. Future research to assess learner performance incorporating manipulation on a 3D model prior to working with subjects for these two prone cervicothoracic techniques may be beneficial. This learning model could potentially improve safety prior to subjects trials. Learners can still receive visual and tactile feedback during instruction which can enhance safety with application of the reviewed techniques prior to subject trials. As described with these two varying techniques provider handling and patient set up can facilitate facet upglide and downglides at intended segments. Therefore, reviewing these techniques can help improve intention of treatment which may be helpful in understanding the osteokinematics with the chosen intervention. The limitations of this study include intersegmental mobility that is depicted on a model and not in vivo and therefore exact representation of zygapophyseal mobility is not exact. The gold standard for assessing intersegmental mobility would be manual techniques performed on a patient under fluoroscopic control. Also, mobility at facet joints is limited and without normal physiological passive and active body subsystem constraints, the motion on a 3D model will be exaggerated. Tissue discrepancy, joint end feel, and patient feedback is a limitation of utilizing the 3D spine model for learning manual techniques. Utilizing this learning model may not be applicable for all HVLA thrust techniques. Lastly, the primary author has extensive experience with application of cervicothoracic HVLA thrust techniques in practice and though this model specifies the procedures and rationale used it does not guarantee that effective manual intervention will be achieved.

## Conclusions

Utilizing a 3D spine model as a learning tool for practicing HVLA thrust techniques may enhance learning for the two reviewed cervicothoracic joint maneuvers. Utilization of this learning model may help facilitate varying aspects of learning and enhance provider rationale for clinical application of manual therapy skills. The cervicothoracic joint HVLA thrust techniques reviewed in this study may help optimize intention to treat for clinicians. Further research is needed to assess learner benefit and performance utilizing this learning model. Further research is warranted to assess the utilization of a 3D spine model for enhancing manual therapy techniques.
